# Herpes Zoster in Four HIV Seropositive Patients and One Patient With Recurrent Carcinoma After Radiotherapy

**DOI:** 10.7759/cureus.21922

**Published:** 2022-02-05

**Authors:** Amirthaleka Muthu Pannerselvam, Jayanthiswari Kulanthaivelu, Karthik Rajaram Mohan, Aadhirai Gopinath, Leo Caroline M

**Affiliations:** 1 Oral Medicine, Mahatma Gandhi Memorial Government Dental Hospital, Tiruchirappalli, IND; 2 Oral Medicine and Radiology, Tamil Nadu Government Dental College and Hospital, The Tamilnadu Dr. M.G.R Medical University, Chennai, IND; 3 Oral Medicine and Radiology, Vinayaka Mission's Sankarachariyar Dental College, Vinayaka Mission's Research Foundation (Deemed to be university), Salem, IND; 4 Orthodontics and Dentofacial Orthopaedics, SRM Dental College, Chennai, IND; 5 Oral Pathology, Chettinad Dental College and Hospital, Chennai, IND

**Keywords:** vesicle, hiv, herpes zoster, gabapentin, acyclovir

## Abstract

Herpes zoster is a ubiquitous ultramicroscopic neurotropic virus that causes pruritic acute grouped vesicular eruptions and rashes, these vesicles rupture spontaneously resulting in pustules, crustations, which are pruritic in nature on the affected skin along the course of the dermatome resulting in scab. The scab withers off later leaving a permanent scar and pigmentation. The characteristic clinical finding was that vesicles or ulcers resulting from herpes zoster lesions never cross the midline. Two such reported cases of herpes zoster in seropositive HIV patients that resulted in extensive crustations and periorbital edema, left unilateral facial pain of burning quality in 25-year-old female patient and spontaneous exfoliation of a tooth in another 35-year-old patient, treated with drug therapy comprising acyclovir, gabapentin, amitriptyline are discussed here.

## Introduction

Viruses are ubiquitous ultramicroscopic microorganisms that cause various infections in human beings [1]. The common viruses causing infection in human beings is a family of herpes viruses [1]. Herpes is a neurotropic virus, which remains latent in the trigeminal ganglion and later expresses itself when conditions are favorable such as underlying immunosuppression like HIV, uncontrolled diabetes, those on long-term corticosteroids after organ transplantation, extreme climates of hot or cold, hormonal changes due to which underlying stress results in menstrual irregularities, and primary infection with varicella resulting in chickenpox during their childhood days [1]. Hereby we report a rare case series of herpes zoster infections that are triggered by HIV infection in four seropositive HIV patients and in one patient with recurrent carcinoma after radiotherapy.

## Case presentation

Case 1

**Figure 1:**
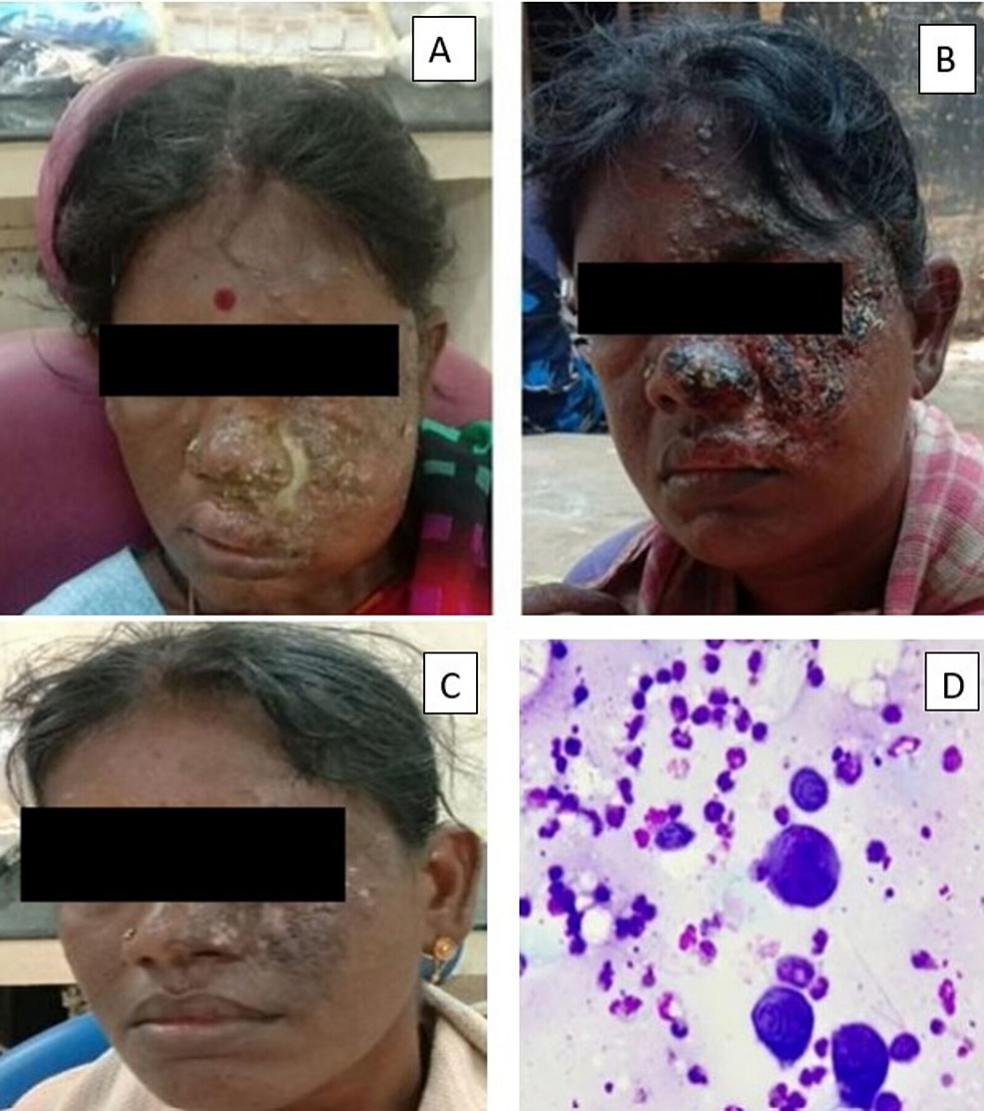
A 35-year-old female patient reported a chief complaint of unaesthetic appearance. A) The female reported a complaint of swelling and discharge in her left side of the face; B) extraoral examination revealed distribution of scabs only on the left side of the face not crossing the midline; C) 1-month follow-up extraoral photograph after anti-retroviral therapy; D) histopathological examination revealed Tzanck cells.

A 35-year-old female patient reported to our dental outpatient clinic with a chief complaint of unaesthetic appearance due to the presence of swelling and discharge in her left side of the face involving the left forehead, left eye, left ala of nose, and left philtrum of the upper lip. On eliciting the history, the patient revealed that she had headache before 2 days for that she applied Zandu pain balm (Emami Group, Mumbai, India), after that she experienced severe burning sensation along with pain on the left side of the face. The patient did not have any prodromal symptoms. On extraoral clinical examination, facial asymmetry was present due to the presence of swelling involving the left cheek and around the left eyelid region. Multiple blisters with pus discharge were present on the left side of the face involving the left forehead region, eye, nose, and left side of the upper lip region, which did not cross the midline of the face. The left eye was completely closed with severe inflammation (periorbital congestion). Pus discharge was present on the left ala of the nose along with vesicular rash. Intraorally blisters with pus discharge were present on the upper labial mucosa. The blisters with pus discharge were unilateral involving the periorbital region, ala and tip of the nose, and upper lip. No rashes were present on the lower lip or mandibular region. By the history, clinical appearance, it was diagnosed as a herpes zoster infection involving v1 (herpes ophthalmicus), v2 (maxillary) branch of the trigeminal nerve on the left side of the face. The patient was sent to the primary healthcare center, Mahatma Gandhi Memorial Hospital, Trichy for an HIV-PCR test. Since she was 35 years old with herpes zoster and well built, her history revealed she was not under any immunocompromised state such as uncontrolled diabetes, but the report came as HIV positive. Her CD4 blood count was 150 cells/mm^3^. Thereby we incidentally figured this case to be of herpes zoster infection due to HIV infection. Simultaneously, COVID-19 test was also done which came as negative. Histopathological examination of fresh vesicle fluid revealed the presence of Tzanck cells under microscopy (Figure 1).

Case 2

**Figure 2:**
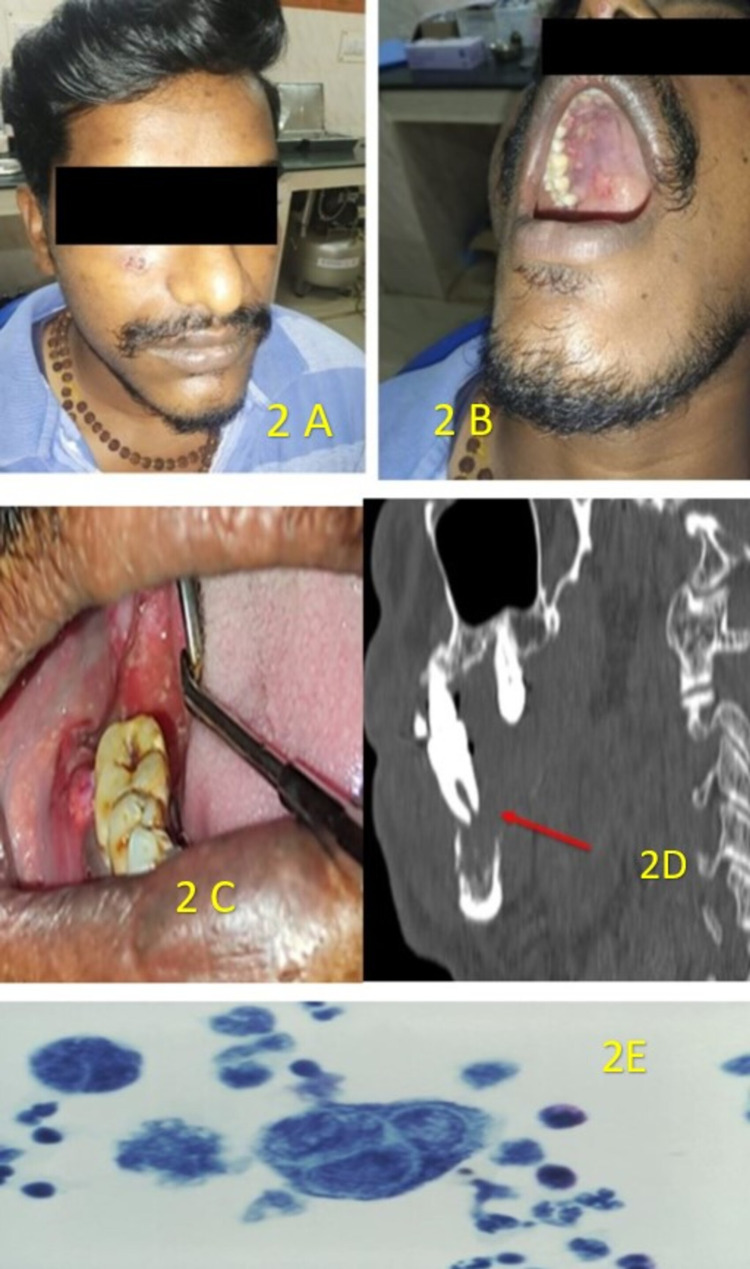
A 25-year-old patient came with a complaint of soreness and pain in his mouth. A) Extraoral examination revealed areas of scabs on the right side of the face; B) intraoral examination revealed numerous crops of ulcers on the right-side lateral half of the hard palate not crossing the midline; C) spontaneous exfoliation of right lower second molar 47; D) sagittal section CT revealed a floating tooth appearance; E) histopathological photomicrograph revealed Tzanck cells.

In the same week, a 25-year-old male patient came to dental outpatient clinic with a complaint of soreness and pain in his mouth for the past 2 days. On eliciting the history, he revealed that the ulcers developed after intake of hot food item 2 days back. The patient was not a diabetic. On extraoral examination, areas of crustation were seen only on the skin of his right cheek region (Figure 2A). On intraoral clinical examination, there were multiple ulcers present only on the right side of the hard palate region which did not cross the midline of the hard palate (Figure 2B). The ulcers were discrete, multiple in number, and irregular in shape, and the margins of the ulcer were erythematous and had sloped edges. There was spontaneous exfoliation of his right lower second molar 47, 3 days back (Figure 2C). The sagittal section of CT revealed the tooth floating in the oral cavity (Figure 2D). Histopathological examination of the peripheral smear of the vesicular fluid revealed Tzanck cells, multinucleated giant cells (Figure 2E).

Based on the above clinical findings, a provisional diagnosis made was herpes infection involving v2 branch of the trigeminal nerve. Histopathological photomicrograph revealed Tzanck cells in peripheral smear (Figure 2E). We suspected this patient also as HIV positive for which he was referred to voluntary health service, while referring the patient, he himself revealed that he was already under antiretroviral therapy.

Case 3

**Figure 3:**
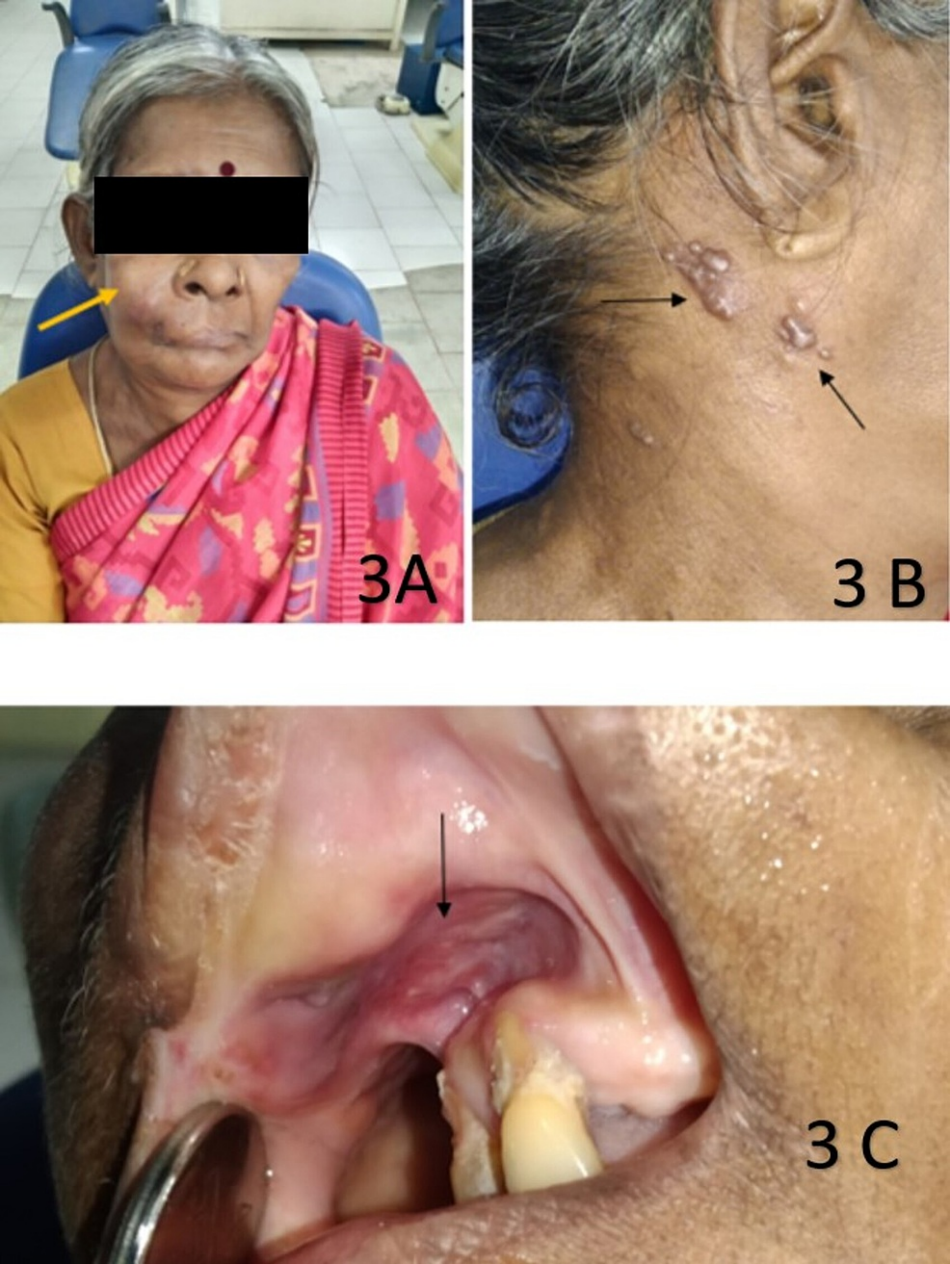
A 72-year-old female reported a chief complaint of swelling and soreness in her mouth. A) extraoral examination revealed swelling on the right side of the face; B) crops of vesicles beneath the tragus of the right ear on the right side of the neck only; C) intraoral examination revealed a non-healing ulcer involving the maxillary labial vestibule.

A 72-year-old female reported a chief complaint of swelling and soreness in her mouth. History revealed the patient was a betel nut chewer for 10 years and was treated by radiotherapy in fractionated dosage of about 2 Gy per week for 7 weeks for carcinoma which occurred in her maxillary labial vestibule region from oral submucous fibrosis. Extraoral examination revealed facial asymmetry due to the presence of swelling on her right side of the face measuring approximately about 5.5 × 4.5 cm in diameter. The swelling extended medially 0.5 cm away from the right side of the ala of the nose, posteriorly 2.5 cm in front of the tragus of her right ear, superiorly extended 1.5 cm away from the right lower eyelid, inferiorly 0.5 cm above her right ala-tragal plane. Obliteration of her right nasolabial fold was present (Figure 3A). The extraoral examination also revealed many crops of vesicles only on the right side of the neck (Figure 3B). Intraoral examination revealed a healing ulcer near her maxillary labial vestibule in relation to the maxillary labial vestibule in the 11, 12, 21 regions (Figure 3C), 13-17, 22-27 regions were missing. Blanched maxillary labial mucosa was present. Her HbA1c level was 8.1 mmol/l, suggestive of poor glycemic control. Correlating the above clinical findings, laboratory report, a final diagnosis of herpes zoster involving C1, C2 dermatome, and recurrent carcinoma involving the maxillary labial vestibule associated with space infection was made.

Case 4

**Figure 4:**
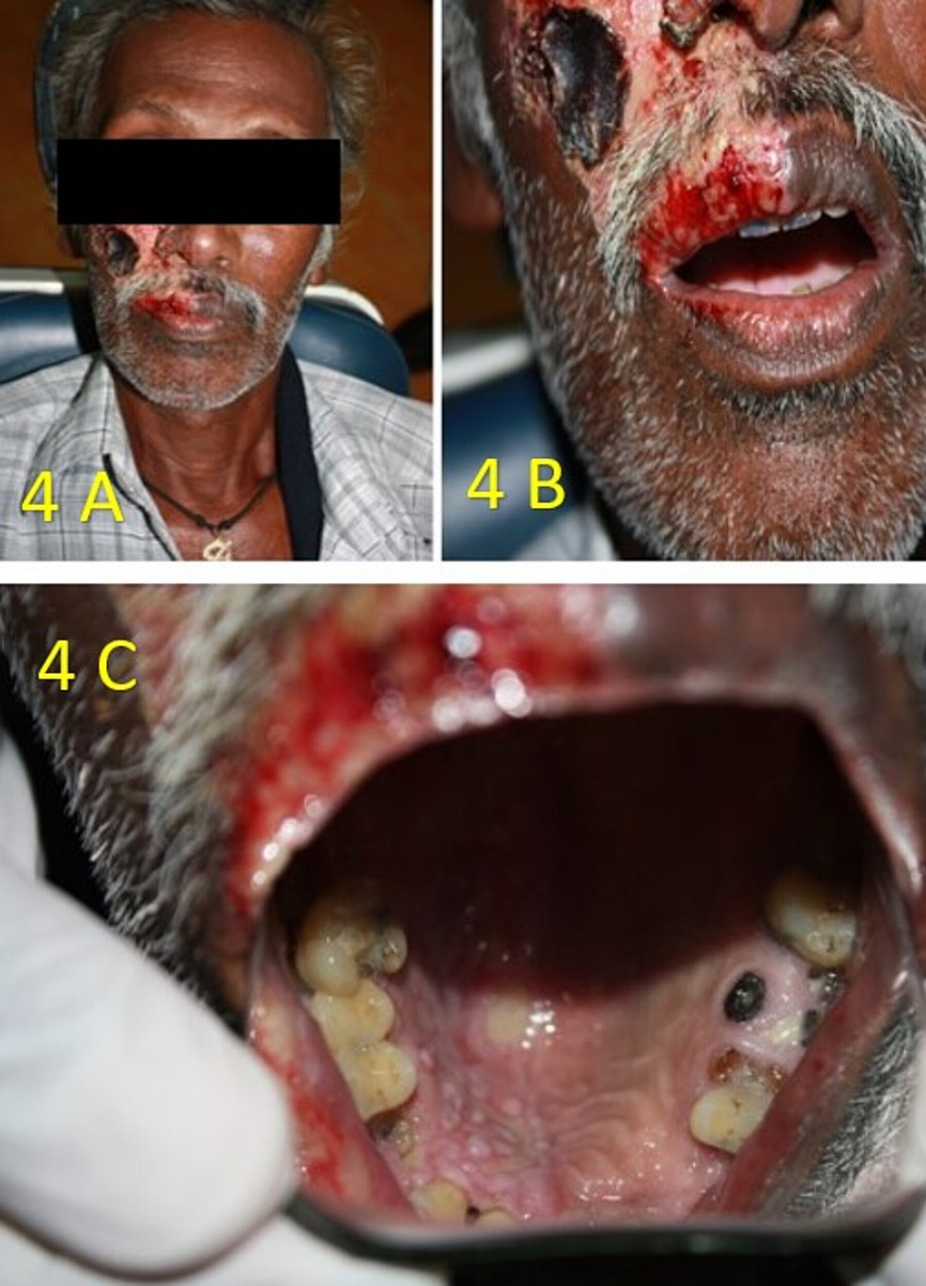
A 67-year-old male reported a chief complaint of severe pain on his right side of the face. A) extraoral examination revealed scalding of the skin on his right side of his face; B) hemorrhagic bloody crustations involving the vermilion border of his upper lip not crossing the midline; C) intraoral examination revealed an ulcer on the right side of the hard palate, not crossing the midline.

A 67-year-old male reported a chief complaint of severe pain on his right side of the face. The patient’s medical history revealed that he was HIV positive. His CD4 blood cell count was 164 cells/mm^3^. He was also recently diagnosed as a diabetic. His blood glucose level was 490 mg/dl and HbA1c level was 10.9 mmol/l, suggestive of poorly controlled diabetes. On extraoral examination, areas of crustations with scalding of the skin were seen only on the right side of his face and bloody crustations involving only on the right vermilion border of the upper lip, not crossing the midline (Figures 4A, 4B). Intraoral examination revealed an ulcer on the right side of the hard palate not crossing the midline (Figure 4C).

Case 5

**Figure 5:**
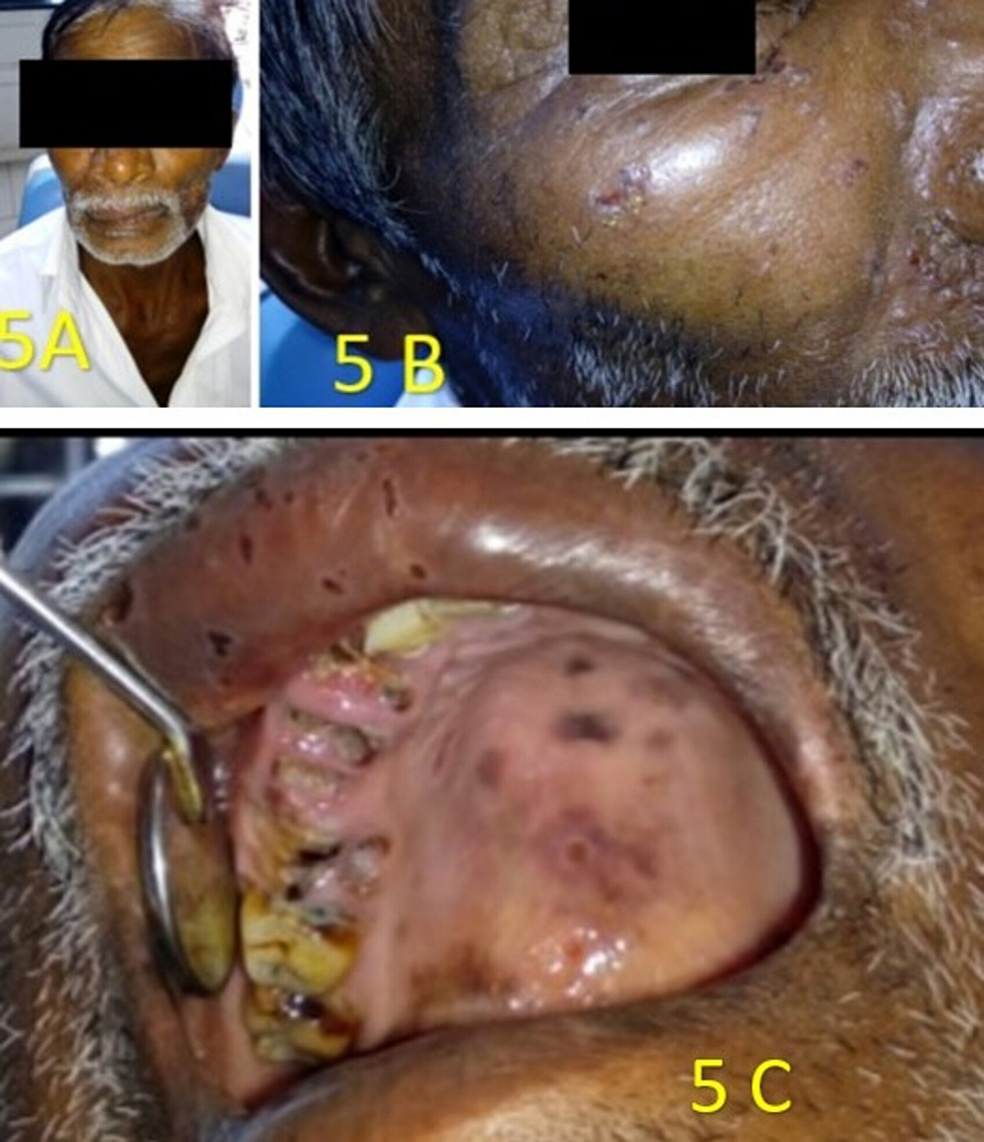
A 53-year-old male reported a chief complaint of pain on the right side of the face. A) numerous crops of vesicles on the right side of the face; B) presence of an intact dew-drop-like vesicle on the skin of right side of the face below the right lower eyelid region; C) intraoral examination revealed discrete areas of scab only on the right vermilion border of upper lip and discrete areas of pigmentation caused by healing of the ulcer that occurred only on the right side of the hard palate, not crossing the midline.

A 53-year-old male reported a chief complaint of pain on the right side of the face. He was positive for HIV. On extraoral examination, an intact dew-drop-like vesicle was seen on the skin on the right side of his cheek and near the right nasolabial fold (Figures 5A, 5B), healed scabs on the right half on the vermilion border of the upper lip (Figures 5A, 5B). Intraoral examination revealed healed areas of greyish pigmented scars on his right-lateral half of the hard palate region, not crossing the midline (Figure 5C). Root stumps were observed in 13-16. Correlating the findings, it was diagnosed as postherpetic neuralgia due to HIV.

Acyclovir 400 mg eight times a day for 1 week was prescribed for both cases 1 and 2 along with prednisolone. An initial loading dose of 60 mg prednisolone per day was given in the first week, followed by 40 mg per day during the second week, 20 mg per day during the third week, and 10 mg during the fourth week along with multivitamin supplements. The female patient was reviewed after 1 week, no fresh blisters were noted and the swelling subsided with scab formation. There were no complaints of vision or problem in hearing and no complaints of difficulty in eating. She had a mild burning sensation over the left half of the face and neck. After 2 weeks, she came for the second review with the complaint of severe pruritis over the left side of the head region. Gabapentin 400 mg was prescribed thrice daily for 1 week and we also referred her to a neuro physician for further management. The male patient did not report for review. All the patients were under antiretroviral therapy namely acyclovir. RT-PCR results for COVID-19 for both the patients came as negative. The third case was prescribed acyclovir 800 mg five times daily for 2 weeks, sitagliptin 50 mg, and metformin 500 mg once daily dosage for her poor glycemic control. Tab. venlafaxine 150 mg once daily at night for 1 week for prevention of postherpetic neuralgia and was also referred to an oncologist for management of recurrent carcinoma of the maxillary labial vestibule.

## Discussion

**Figure 6:**
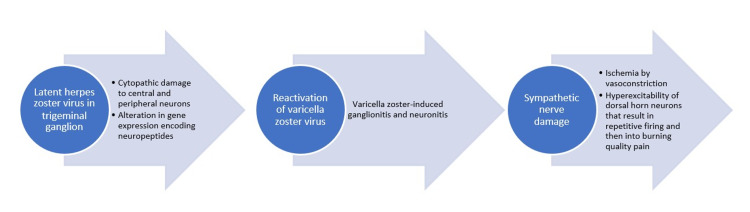
Etiopathogenesis of herpes zoster.

Herpes zoster is a viral infection, caused by the reactivation of latent virus varicella-zoster that remains dormant in the trigeminal ganglion. Herpes is also known as shingles. The term “herpes” literally means “to creep or crawl” denoting the spreading lesions. Herpes is a neurotropic virus that can cause axonal degeneration resulting in the transmission of latent virus and causing sensorineural damage which ultimately results in loss of sensation in the affected dermatome. The etiopathogenesis of herpes zoster infection is described in Figure 6.

**Figure 7:**
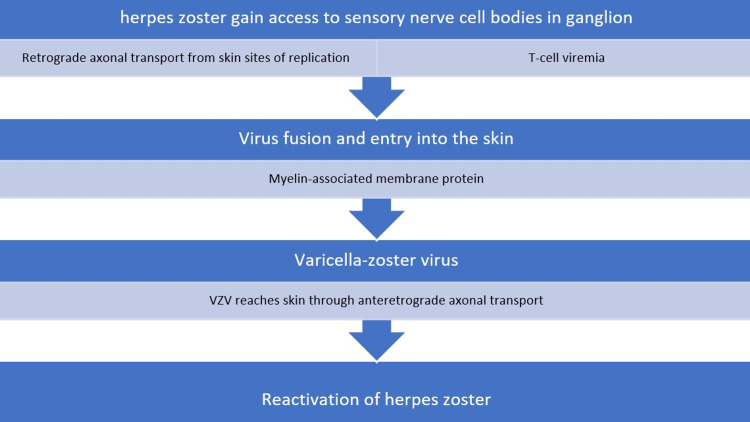
Genomic transmission of herpes zoster. VZV: varicella-zoster virus

The genomic transmission of herpes zoster is described in Figure 7.

The triggering or precipitating factors for herpes zoster include immunocompromised individuals such as patients with poorly or uncontrolled diabetes, patients with malignancies who have altered coagulopathy, patients having long-term chemotherapy or radiation therapy for cancer, patients on immunosuppressants after an event of organ transplantation, extreme climatic variations like traveling under too hot or too cold climate, and females having menstrual periods with mood swings or acute depression. Herpes zoster is characterized by a prodromal period with burning pain for 2-3 days, followed by a vesicular eruption in the dermatomal distribution of infected trigeminal ganglion. The most commonly affected dermatomes include T1 to L2 (thoracic, followed by cranial (especially trigeminal), lumbar, cervical, and sacral (least affected dermatome)). The pain usually can be severe and burning in quality that interferes with sleep. Initially results in a rash followed by the appearance of vesicles in 1 or 2 days. Pustules follow within a week after the rash later results in ulceration and crust and finally lead to scab that gets withered away. The vesicles rapidly burst and spread to the adjacent site along the dermatome but never cross the midline resulting in unilateral dermatomal rashes. Usually, the pain starts along the course of the nerve initially followed by multiple grouped vesicular eruptions, which rupture to form crustations and scabs. The dental complications of herpes zoster infection include odontalgia due to localized vasculitis resulting in the infarction of trigeminal nerve and vessels and avascular necrosis resulting in irreversible inflammation of pulp followed by pulpal necrosis, internal resorption, exfoliation of teeth, postherpetic neuralgia, and intractable facial pain that persists for about 3 months following the infection [1-3]. Lesions are usually on the face and trunk. HIV-infected patients are 20 times more likely to develop herpes zoster, often before other clinical findings of HIV disease are present. Disseminated herpes zoster usually is defined as a generalized eruption of more than 20 extra-dermatomal vesicles occurring within a week of the onset of classic dermatomal herpes zoster [4,5].

A history of HIV risk factors and HIV testing when appropriate should be considered, especially in patients with herpes zoster who are younger than 55 years. Here, we discussed a case of herpes zoster involving an ophthalmic and maxillary branch of the trigeminal nerve on the left side in a 35-year-old female patient (Case 1) and maxillary division of trigeminal nerve in a 25-year-old male patient (Case 2), who was on antiretroviral therapy. The female patient (Case 1) was presented with unilateral vesicular rashes and pus discharge involving the left forehead region, eye, nose, and upper lip region. Periorbital congestion was present. Since the ala and tip of the nose were involved Hutchinson’s sign was positive in our case and this indicates the involvement of the nasociliary branch of the ophthalmic nerve. There was no blurring or blindness in her vision, she had mild conductive hearing loss. The vesicles were limited to the upper lip region. The patient did not have any prodromal symptoms like fever but she had complaints of severe burning sensation limited to the left side of the face. We diagnosed this case as herpes zoster infection of the left side of the face involving the course of the trigeminal nerve (v1 and v2). Often herpes zoster occurs in patients in immunodeficient states, since the patient was young and well built without any other systemic illness we referred her to check for COVID-19 and HIV-RNA-PCR test, the result came as HIV positive and COVID-19 negative. We prescribed acyclovir 800 mg five times a day for 1 week along with gabapentin (Tab. gabafine 300 mg) thrice daily. A tricyclic antidepressant Tab. amitriptyline 75 mg was prescribed once daily at night for 7 days. The ruptured vesicular areas with crustations on the left side of the face in the female patient markedly reduced, the periorbital inflammation involving the left eye drastically reduced and, the burning quality of pain on her face reduced. The male patient (Case 2) did not turn up for review. Various previous case studies of herpes zoster in HIV patient are enumerated in Table 1.

**Table 1:** Research studies on cases of herpes zoster

Author	No of cases reported	Year	Age/sex	Chief complaint	Oral manifestations
Cloarec et al. [5]	1	2014	50-year-old male	Skin rash on the face and purulent discharge from the ear. the patient experienced spontaneous, multiple (five) and nonalgic tooth exfoliation	Ulceronecrotic gingivitis in right mandible associated with bone exposure and no bleeding at contact. Teeth 43 and 44 were mobile and were finally spontaneously exfoliated a few days later
Grillo et al. [6]	1	2013	54-year-old female	Odynophagia, pharyngeal swelling, painful skin lesions on her left ear	Circumscribed erosions on the left lateral anterior two-third of the tongue and left palate
Borumandi [7]	1	2013	43-year-old male	Non-healing wound after extraction of upper and lower right third molars 6 months earlier	Wound dehiscence with exposed necrotic alveolar bone on the adjacent tooth, while the lower right second molar was still vital and immobile
Lutwak and Dill [8]	1	2012	68-year-old male	3 days of a painful rash on his left upper back and left arm	Herpes zoster is common among patients with HIV
Shin et al. [9]	1	2010	51-year-old male	Disseminated herpes zoster usually is defined as a generalized eruption of more than 20 extradermatomal vesicles occurring within a week of the onset of classic dermatomal herpes zoster	Disseminated herpes zoster may be the first manifestation of HIV infection
Rajashekar et al. [10]	1	2008	30-year-old male	Multiple, painful, fluid-filled skin lesions of 8 days’ duration	Involvement of the same dermatome (T-10) has recurred on the contralateral side (duplex symmetricus)
Omoti and Omoti [11]	1	2007	32-year-old female	Vesiculopapular rashes with hyperpigmented crusts over the maxillary area of the face on the left side with periocular edema, conjunctivitis, and mild punctate keratitis in the left eye	
Meer et al. [12]	1	2006	70-year-old diabetic male	“pins and needles” sensation in his face and “toothache” in the left mandible	Extensive necrotizing gingivitis in the lower left quadrant with marked mobility of teeth #18 to #23 Bone was exposed distal to the lower left second premolar and pus was noted draining from the gingival margins of the teeth
Mendeita et al. [13]	1	2005	63-year-old female	Pain of several weeks’ duration in the upper right lateral incisor	Redness of the alveolar mucosa and gingiva of the lower right quadrant with multiple well-delimited and painful erosive lesions affecting the attached gingiva around the teeth and loosening of teeth
Siwamogstham et al. [14]	Total=4	2002-June	30-year-old female	Pain on the upper left lateral incisor and canine for a period of 3 months	Loosening of teeth, gingival bleeding, pseudomembranous candidiasis on the buccal mucosa, dorsum of tongue, and sublingual space. Exposure of necrotic alveolar bone
		1998 -February	31-year-old Thai man	Complaint of extensive ulceration and scarring with hyperesthesia of the right side of the face, which had been present for 1 month	Generalized gingivitis with moderate calculus formation especially on the lower right side. The lower right central incisor had just exfoliated spontaneously, painlessly leaving a non-healing socket (Figure 2B). The alveolar bone was exposed with severe mobility of teeth involving the apical third of the labial side of the lower left central incisor and lingual side of the lower right first molar
		1995-June	29-year-old-Thai man	Complaint of a painful lesion on the right buccal mucosa	Intraoral examination revealed poor oral hygiene with generalized gingivitis. The gingival area of the mandibular right second premolar was red and swollen. There was excessive loosening of the lower right first premolar and second molar. The second premolar had exfoliated spontaneously without pain leaving a non-healing wound with a necrotic alveolar socket. The retained root of the lower right first molar was covered by inflamed gingiva (Figure 3A). The buccal plate from the lower right first premolar to the second molar was exposed and necrotic
		1995-May	31-year-old female	Complaint of extreme mobility of her lower left teeth	Severe loosening of the lower left central incisor, canine, second premolar, and first, second, and third molars. The lower left lateral incisor and first premolar had exfoliated spontaneously because of total loss of the bony socket 2 days prior to admission leaving raw and necrotic bone walls. The left mandibular alveolar labial and lingual plate from the canine to the mesial wall of the lower third molar were exposed and necrotic. There was no gingival tissue surrounding these teeth. All lower left teeth showed a positive electrometric pulp response. Generalized gingivitis

Glesby et al. stated that herpes zoster can occur at all CD4 count levels in HIV-infected adults [15]. Saguil et al. stated that acyclovir must be initiated within 48-72 hours to prevent postherpetic neuralgia in herpes zoster-infected patients [16]. Wassilew et al. in their study suggested the use of oral brivudine (125 mg) once daily for 10 days for effective management of postherpetic neuralgia, an intractable facial pain that occurs during the development or healing of rash in herpes zoster patients [17]. History of hidden diabetes must also be screened in patients with herpes zoster viral infection.

## Conclusions

Whenever a patient complains of facial pain and unilateral ulcers on the hard palate not crossing the midline, a history of HIV risk factors and HIV testing on admission must be considered especially in patients younger than 55 years. Whenever the patient reports viral infections like herpes zoster, it is essential to go for investigations like the HIV-RNA-PCR test and COVID-19 and we must also be aware that herpes zoster can occur in immunocompromised individuals affected by recurrent carcinoma. The rare occurrence of herpes zoster along a cervical dermatome (C1, C2), which is least affected, is presented. Literature reveals that there is a possibility of herpes infections in corona patients, HIV, and other immunocompromised states due to uncontrolled diabetes and carcinoma. We, the oral physicians, must be aware of the clinical presentation to rule out these viral infections to provide correct management of the patient, and to prevent further life-threatening complications. By prompt antiviral therapy as soon as herpes zoster infection is suspected, the dangerous complications of sudden blindness and postherpetic neuralgia are also prevented in these patients.
